# A Relational Framework for Artificial Intelligence-Assisted Shared Decision Making

**DOI:** 10.3390/healthcare14213645

**Published:** 2026-10-28

**Authors:** Gianmarco Di Palma, Anna De Benedictis, Flavia Giacomobono, Davide Ferorelli, Vittoradolfo Tambone, Roberto Scendoni, Francesco De Micco

**Affiliations:** 1 Operative Research Unit of Clinical Affairs, Fondazione Policlinico Universitario Campus Bio-Medico, 00128 Rome, Italy; g.dipalma@policlinicocampus.it (G.D.P.);; 2 Research Unit of Bioethics and Humanities, Department of Medicine and Surgery, Università Campus Bio-Medico di Roma, 00128 Rome, Italy; 3 Research Unit of Nursing Science, Department of Medicine and Surgery, Università Campus Bio-Medico di Roma, 00128 Rome, Italy; 4 Interdisciplinary Department of Medicine (DIM), Section of Legal Medicine, University of Bari “Aldo Moro”, 70121 Bari, Italy; 5 Department of Law, Institute of Legal Medicine, University of Macerata, 62100 Macerata, Italy

**Keywords:** artificial intelligence, shared decision making, informed consent, patient–clinician relationship, clinical governance, human oversight, uncertainty, health equity

## Abstract

**Background:** Artificial intelligence (AI) can support clinical decision making, but accuracy, usability, and efficiency do not establish whether patients understand options or influence the agreed plan. This study aimed to develop observable relational requirements for AI-assisted shared decision making. **Methods**: We conducted a qualitative framework synthesis of 21 clinical, empirical, conceptual, and governance sources identified through structured purposive sampling completed on 20 September 2026. Two-pass coding combined established shared decision-making categories with inductive analysis of AI-related relational effects. Domains were retained when supported across source types, conceptually distinct, observable at the point of care, and linked to a clinical or organizational response. **Results**: The synthesis identified a patient–clinician–AI interaction embedded in organizational governance and highlighted five relational concerns: apparent authority of AI output, insufficient verification and contestability, substitution of inferred for expressed preferences, uncertainty without actionable follow-up, and fragmented responsibility. These concerns informed five domains: role and influence disclosure; independent verification and contestability; direct preference elicitation; actionable uncertainty and revisability; and distributed accountability and continuity. Each domain was translated into a relational question, a clinical test, and a documentation or governance response. A complementary measurement framework distinguished technical performance from decision process, relational quality, equity, safety and continuity, and governance outcomes. **Conclusions**: The study’s contribution is an operational framework connecting AI-related relational concerns to observable clinical requirements and candidate evaluation measures. The proposed framework requires stakeholder and prospective clinical validation; it does not establish clinical effectiveness.

## 1. Introduction

### 1.1. AI in Shared Decision Making: Opportunities and Relational Challenges

Shared decision making is a clinical process in which a patient and a health professional recognize that a decision exists, compare reasonable options, discuss the consequences that matter, and agree on a plan that reflects the patient’s informed preferences [1]. Predictive models estimate outcomes, decision aids organize alternatives, generative systems produce explanations, and ambient systems summarize encounters or reduce documentation work. These functions differ in purpose and risk, but all can affect which information becomes visible, how uncertainty is framed, and which recommendation appears authoritative. Evidence to date supports cautious interest rather than a general claim that artificial intelligence improves shared decision making. A systematic review of artificial intelligence in collaborative care planning included 39 publications identified through a search completed in March 2024. The review combined trials, predictive model studies, protocols, qualitative research, and conceptual work, which precluded a pooled estimate of effect [2]. A second systematic review included 26 studies of patient and clinician perspectives on artificial intelligence-enabled decision aids. Patients often described the tools as understandable and useful, while clinicians raised concerns about outdated information, omitted risk factors, bias, and over-treatment or under-treatment [3]. The perceived usefulness of an interface and the quality of a decision therefore remain different outcomes. In a pragmatic randomized trial involving 238 outpatient physicians, one ambient scribe reduced time spent on notes, while a second system did not produce a statistically significant reduction; occasional clinically significant inaccuracies were also reported [4]. Reduced documentation burden may create time for care, but it improves the patient–clinician relationship only if the time is used for listening, explanation, verification, or follow-up. Artificial intelligence also changes the distribution of information. Patients increasingly use generative systems before or after consultations. Such tools can translate reports, help prepare questions, and provide a coherent account of a condition. They can also fabricate sources, overlook comorbidities, and express population estimates as confident individual advice. Hack and colleagues describe the AI-educated patient and propose soft accountability, in which a better prepared patient may prompt a clinician to explain decisions more fully, while emphasizing that this mechanism remains a hypothesis [5]. The same interaction may increase conflict when a fluent but incorrect answer has already shaped the patient’s expectations. These developments extend a concern already identified in analyses of person-centred care: an artificial intelligence system can influence the encounter without becoming a moral or legal agent [6]. Numerical precision, fluent language, and integration into the health record can confer authority even when the output requires interpretation. In interviews with 18 patients, eight health professionals, and eight developers about a postpartum depression risk model, participants wanted actionable information, flexible communication, professional interpretation, and clearer responsibility for errors and follow-up [7]. A scoping review of 67 studies on artificial intelligence for measuring or predicting patient values found promising technical performance alongside limited real-world validation, representativeness problems, and a tendency to predict preferences for decision support rather than help patients understand and express their own preferences [8].

### 1.2. Existing Guidance and Rationale for the Framework

The ethical question is therefore not whether artificial intelligence can contribute information, but how its contribution supports patient participation while preserving professional judgment and organizational accountability. CONSORT-AI and SPIRIT-AI address transparent reporting of AI intervention trials and their protocols, including human–AI interaction [9,10]. DECIDE-AI addresses early-stage clinical evaluation, with explicit attention to human factors, safety, and clinical integration [11]. WHO guidance provides ethical principles and governance recommendations for AI in health, including large multi-modal models [12]. These documents are not considered inadequate within their intended scope. The present study addresses a more specific operational question: how to organize relevant safeguards into observable requirements for an AI-assisted shared decision-making encounter and its supporting care pathway. The proposed contribution is to connect disclosure of AI influence, verification and contestability, direct preference elicitation, actionable uncertainty, and accountability with clinical tests, documentation responses, and candidate outcomes.

### 1.3. Study Objective

This study aimed to develop a complementary framework through a qualitative synthesis of clinical, empirical, ethical, legal, and governance sources; it does not establish that existing guidance is ineffective or that the proposed framework is superior.

## 2. Materials and Methods

### 2.1. Study Design

We conducted a qualitative framework synthesis and methodological development study. The applied research question was: Which observable relational conditions must be present for an artificial intelligence-assisted clinical decision to remain meaningfully shared? The framework method and directed qualitative content analysis support systematic comparison across heterogeneous cases while allowing deductive categories and inductive themes to be combined [13,14]. The intended output was an operational framework linking each relational requirement to a clinical test, a documentation or governance response, and candidate outcomes for validation. The synthesis integrated empirical and normative evidence because the research question concerns both what occurs in practice and what patients, clinicians, and organizations should be able to understand, challenge, and document. The study aimed to develop a framework for subsequent evaluation; it did not test prespecified hypotheses, estimate intervention effects, or assess the framework’s validity or clinical effectiveness. Legal and professional sources were interpreted within their stated jurisdictions and functions rather than treated as empirical observations.

### 2.2. Source Identification and Eligibility

Sources were identified through searches of PubMed, Scopus, and Web of Science using a structured purposive sampling strategy, completed on 20 September 2026. Sampling followed five complementary streams: foundational models of shared decision making; systematic or scoping reviews of artificial intelligence and decision support; empirical studies that examined use, implementation, training, or stakeholder experience; conceptual and professional analyses of the patient–clinician relationship; and official legal, regulatory, professional, or governance instruments. Evidence syntheses were used to map the field, while maximum variation sampling added sources addressing technologies, clinical contexts, stages of the patient journey, and governance levels that were not adequately represented by the reviews. Official instruments were obtained from the issuing authority or an authoritative publication source. A source was eligible when it was available in full text in English or Italian, concerned healthcare, and contributed directly to at least one prespecified question: what function artificial intelligence performed; who encountered or acted on the output; how the output affected information or choice; which relational or ethical risk arose; which safeguard was proposed; or which outcome could be evaluated. We excluded purely technical performance reports with no link to patient participation, professional judgment, implementation, or governance; non-healthcare applications; promotional material without an identifiable evidentiary or normative basis; duplicates; and sources for which the relevant claims could not be verified in the full text. The final analytical corpus contained 21 sources: two foundational shared decision-making frameworks [1,15], three evidence syntheses [2,3,8], three empirical studies [4,7,16], seven conceptual or professional analyses [5,6,17,18,19,20,21], and six legal, regulatory, or governance sources [12,22,23,24,25,26]. Categories describe each source’s primary analytical contribution and are not mutually exclusive. Methodological references supporting the analytical approach [13,14] were not counted as corpus sources. The corpus size was not predetermined. A complete screening log was not maintained; therefore, the total number of records considered and a numerical breakdown of exclusions cannot be reported. Table 1 summarizes the sources included in the analytical corpus and their primary contributions.

### 2.3. Data Charting and Coding

The unit of analysis was a statement linking an artificial intelligence function to a consequence for patient participation, clinician judgment, or organizational accountability. Each statement was charted across six fields: actor, system function, stage of the patient journey, relational effect, proposed safeguard, and candidate outcome. Actors included patients, trusted persons, clinicians, other health professionals, developers, providers, and healthcare organizations. System functions included prediction, classification, ranking, summarization, language generation, documentation, and preference inference. Source appraisal was appropriate to source type. For evidence syntheses, we recorded review scope, number and type of included studies, search period, and stated limitations. For empirical studies, we recorded design, participants, setting, outcomes, and limitations relevant to transferability. For legal, regulatory, and professional materials, we recorded issuing authority, jurisdiction, legal or advisory status, scope, and date in force. Conceptual analyses were assessed for clarity of argument, stated assumptions, and connection to clinical practice. This appraisal informed the strength and scope of each claim; heterogeneous source types were not collapsed into a common numerical quality score. Coding proceeded in two passes. The first pass used deductive categories from shared decision making: recognition of a decision, comparison of options, elicitation of preferences, deliberation, and agreement on a plan [1,15]. Governance categories included autonomy, safety, transparency, accountability, inclusion, and equity [12]. The second pass applied inductive coding to effects not captured adequately by those categories, including apparent authority created by fluent language, displacement of direct preference elicitation by inferred preferences, disagreement between clinician and system, inhibition associated with recording, and the need to translate uncertainty into follow-up. Codes were charted in a source-by-domain matrix and compared across technology types, clinical contexts, patient-journey stages, and levels of responsibility. Discordant material was retained rather than averaged. Examples included favorable usability assessments alongside concerns about outdated information or bias, efficiency gains alongside clinically significant inaccuracies, and potential value in preference prediction alongside objections to treating inferred preferences as expressed wishes. Frequency of a code was not interpreted as evidence of ethical or clinical importance.

### 2.4. Framework Construction and Credibility

The framework was constructed in three analytical passes. The first mapped how artificial intelligence changed the informational structure of the encounter. The second translated recurrent safeguards into clinical tests applicable to clinician-facing systems and patient-supplied artificial intelligence content. The third linked each test to measurable outcomes and organizational prerequisites. A candidate domain was retained only when it met four criteria: support from more than one source type; conceptual distinction from adjacent domains; expression as an observable question at the point of care; and a corresponding clinical, documentation, or governance response. The transition from codes to domains involved grouping related relational effects and safeguards according to their primary function in the decision-making process. Each resulting domain was formulated as a relational question and linked to a clinical test, a documentation or governance response, and candidate evaluation measures. Credibility was strengthened through source-type triangulation, explicit preservation of discordant findings, and negative-case testing. The proposed domains were examined against end-of-life decisions, preference prediction, risk estimates derived from under-represented populations, ambient recording, and unverified generative output. An audit trail linked each domain to source statements, safeguards, and candidate outcomes. Because coding and framework development were performed by one author, the final framework is presented as a testable proposition rather than a validated consensus standard.

### 2.5. Ethical Considerations

The study analyzed published literature, legislation, professional standards, and public guidance. It did not involve patients, research participants, identifiable health data, or confidential organizational data. Institutional review board approval and informed consent were therefore not applicable.

## 3. Results

### 3.1. Characteristics of the Evidence Base

The 21-source corpus covered predictive models, AI-enabled decision aids, generative systems, ambient documentation, and methods that infer patient values or preferences. Clinical contexts included screening, prevention, surgery, maternity care, oncology, primary care, medical education, and end-of-life decision making. The sources addressed different outcomes and could not support a common effect estimate. Only a minority directly measured the quality of the shared decision process. Many evaluated accuracy, usability, perceptions, workload, or educational confidence instead. The three evidence syntheses illustrate this gap. The 39-publication review of collaborative care planning contained empirical, protocol, and conceptual work [2]. The 26-article review of user perspectives focused on understandability, usefulness, bias, access, anxiety, privacy, and risks of over-treatment or under-treatment [3]. The 67-study scoping review of patient values and preferences reported that many applications predicted preferences from population or individual data, but few were designed to help patients reflect on their own priorities; most applications remained theoretical or proof of concept with limited external validation [8]. The empirical studies added practical constraints. The ambient scribe trial separated time outcomes from accuracy concerns [4]. The educational pilot showed that simulation could combine interpretation of artificial intelligence output with option discussion and patient feedback, while its small uncontrolled design did not establish patient benefit or error reduction [16]. The stakeholder interview study identified a demand for actionable outputs, professional explanation, adaptable communication, stakeholder involvement during development, and shared responsibility across roles [7].

### 3.2. A Triadic Encounter Embedded in an Organizational System

Artificial intelligence changes the patient–clinician relationship into a triadic interaction. The patient contributes lived experience, goals, tolerance of risk, and practical constraints. The clinician identifies clinically appropriate options, interprets evidence, and accepts responsibility for the recommendation. The system organizes data, estimates outcomes, generates language, ranks alternatives, or records the encounter. It does not need moral or legal agency to affect the decision because it can change what both parties see and the authority they attribute to a recommendation. The triad sits within an organizational system. Procurement, configuration, data quality, integration, access controls, version management, performance monitoring, training, and incident response shape what occurs in the consultation. A clinician cannot exercise meaningful oversight if the organization provides neither the information nor the time needed to inspect the output. The clinical relationship is therefore the point at which system effects become visible, but it is not the only level at which responsibility resides.

### 3.3. The Five Domains of the Relational Framework

#### 3.3.1. Role and Influence Disclosure

Transparency must explain the system’s function and its material influence on the proposal. The patient needs to know whether the system interpreted an image, estimated a risk, ranked options, summarized the record, drafted educational material, or documented the encounter. Technical detail is relevant when it changes understanding, but a description of model architecture can obscure the central issue if the patient still cannot identify what the system contributed or who judged the result. Material influence determines the depth of disclosure. An unnoticed spelling aid and a risk estimate that changes the recommended treatment do not require the same conversation. Useful disclosure connects the system to the choice: what information it added, whether it altered the proposal, which limitation matters in this case, and how a professional reviewed the result. Italian Law 132 of 2025 recognizes a right to information about the use of artificial intelligence in healthcare and places the decision with medical professionals [23]. Read together with Law 219 of 2017, this supports meaningful explanation within the consent process rather than a generic technology notice [22]. Within the proposed framework, AI influence is material when its output is used as a reason for selecting, excluding, or prioritizing a clinical option, or for determining the urgency or follow-up of care. The clinician can assess this by identifying whether, and how, the output contributed to the recommendation presented to the patient.

#### 3.3.2. Independent Verification and Contestability

Clinically relevant output requires verification that is independent of the system’s own fluency or confidence. The clinician checks the inputs, intended use, definition of the output, source evidence, validation setting, omissions, and consistency with the history, examination, and other findings.

In a vignette-based evaluation of ten large language models, asking “Are you sure?” without providing additional clinical information was followed by lower aggregate diagnostic accuracy, with more correct-to-incorrect than incorrect-to-correct revisions [27]. These findings support distinguishing a model’s reconsideration of its own answer from independent verification against external evidence. Contestability includes the ability to question both the result and the objective that produced it. A system prompted from the perspective of an insurer, institution, clinician, or patient may privilege different outcomes even when the clinical facts are unchanged. The relevant question is which objective the system optimizes and whether that objective is clinically appropriate and aligned with the patient’s interests. Disagreement between clinician and system is a diagnostic signal. The appropriate response is to examine inputs, evidence, missing features, and intended use, then seek an alternative method or second opinion when the stakes justify it.

#### 3.3.3. Direct Preference Elicitation

Prediction and preference answer different questions. A model estimates an outcome under specified conditions. Shared decision making asks what should be done when the consequences of reasonable options matter differently to this person. A favorable predicted outcome may be a poor guide when the model emphasizes an endpoint the patient values less, uses an irrelevant time horizon, or overlooks the burden of treatment, work, caregiving, mobility, or daily independence. Artificial intelligence may help identify possible priorities, organize preference data, or prompt questions. It must not replace a patient’s current account when the patient can participate. Inferred preferences can be incomplete because demographic variables, previous choices, electronic records, and digital traces reflect past contexts and may not capture changing values. The 2025 scoping review found that preference prediction frequently served clinicians or service optimization, while few studies helped patients understand their own preferences [8]. In end-of-life decisions, the SIAARTI Ethics Committee warned that digital traces and recorded conversations cannot automatically assume the status of an expressed wish and may conflict with family narratives or the evolving meaning of earlier statements [20].

#### 3.3.4. Actionable Uncertainty and Revisability

Uncertainty arises from incomplete inputs, population differences, model drift, calibration, changing clinical conditions, and the limits of individual prediction. It also arises from deliberation: a patient may need time to understand which consequences are acceptable. More detailed prediction does not resolve uncertainty about values. A number should be used only after its meaning is established. A score of 0.82 is not necessarily an 82 percent probability. A probability requires a defined outcome, time horizon, reference population, and evidence that predicted risks correspond to observed frequencies. Risk communication should support fair comparison through consistent outcomes and time horizons, baseline risk, and absolute effects where possible. Confidence intervals or uncertainty statements should not be invented when they are unavailable. The relational requirement is to connect uncertainty to action. Within the proposed framework, uncertainty is made actionable when the unresolved issue is linked to an agreed response, with an identified responsible professional or service and a timeframe or clinical trigger for review. The patient should know what remains unknown, whether additional information is needed, when the decision will be revisited, which symptoms or changes should prompt earlier contact, and who will respond. Recent stakeholder interviews show why actionability matters: patients and professionals may accept that a model predicts risk yet remain unsure what a threshold means or what to do next [7]. A revisable plan prevents a probabilistic output from closing discussion prematurely.

#### 3.3.5. Distributed Accountability and Continuity

Human oversight is superficial when the designated professional lacks training, time, access to system information, or authority to challenge the output. Bedside responsibility and organizational responsibility are connected but not interchangeable. Clinicians must use authorized systems appropriately, protect confidentiality, verify clinically important outputs, explain material effects, and document their reasoning. Organizations must select and configure systems, govern data, monitor performance, manage versions, provide training and technical support, and maintain incident reporting and escalation pathways [12,17,18,24]. Accountability should follow the actual chain of decisions. A harmful output does not by itself identify the responsible actor. Review must reconstruct the system’s function, available information, permitted controls, mode of use, organizational safeguards, and clinical consequences. The immediate priorities are patient protection, candid communication about established facts, preservation of relevant evidence, and appropriate escalation. Documentation should identify the material contribution of artificial intelligence, the clinician’s interpretation, the patient’s preferences, unresolved uncertainty, and the agreed follow-up without copying unnecessary sensitive content into the record.

#### 3.3.6. Rationale for the Five-Domain Structure and Cross-Cutting Themes

The five-domain structure organizes the synthesis around five distinct questions about an AI-assisted decision: What influence did the system have on the recommendation? How was its output checked and challenged? Whose preferences informed the choice? How will uncertainty affect action and reassessment? Who is responsible for implementation and follow-up? These questions distinguish informational transparency, epistemic scrutiny, preference formation, management of uncertainty over time, and responsibility across the care pathway. Support across source types was a corroborative criterion rather than a sufficient justification for a separate domain. Conceptual distinction, observability, and an identifiable clinical or organizational response were also required. The boundaries between domains concern their primary function rather than complete independence: explaining AI involvement does not establish that its output was verified; verifying an estimate does not establish that the patient’s priorities informed the decision; and agreeing on a plan does not establish responsibility for reassessment or follow-up. Privacy and equity remain essential but operate across these functions. Privacy affects authorized use, disclosure, documentation, and accountability; equity affects accessibility, the applicability of AI output, preference elicitation, and continuity. Calibrated trust is treated as an outcome to evaluate rather than a separate procedural requirement. Five domains are therefore proposed as a clinically interpretable organization of the synthesis, not as an empirically optimal number or an exhaustive taxonomy. Alternative structures remain possible, and stakeholder assessment is needed to determine whether domains should be merged, divided, or supplemented.

### 3.4. Application Across the Patient Journey

The five domains apply before, during, and after the encounter. Before the visit, artificial intelligence may identify a decision, prepare questions, or expose the patient to a recommendation. During the encounter, it may display predictions, structure alternatives, generate explanations, or record the conversation. After the visit, it may summarize the plan, answer follow-up questions, monitor symptoms, or generate reminders. The relational risk changes by stage, so a single consent event cannot govern the entire pathway. Table 2 and Table 3 provide complementary views: Table 2 links each domain to a clinical test and a documentation or governance response, while Table 3 locates the corresponding risks and safeguards along the patient journey. Consider a patient who brings an AI-generated treatment recommendation to a consultation. Before deliberation, the clinician identifies its source and limitations and clarifies that the choice remains open, addressing false confidence and premature closure (Table 3). During the encounter, the clinician checks the recommendation against clinical findings and external evidence, compares reasonable alternatives, and elicits the patient’s priorities (Table 2). The record captures why the recommendation was accepted, modified, or rejected and which preferences informed the plan. After the encounter, any generated summary is checked against that plan, and follow-up responsibility and reassessment triggers are specified, addressing the continuity risks in Table 3.

### 3.5. Measurement Framework

The intended purpose of shared decision making determines what should be measured. Discrimination, calibration, error rates, and processing time remain necessary system outcomes. They do not show whether the patient understood the options, expressed preferences, or influenced the plan. The proposed measurement framework therefore separates five outcome levels while preserving their relationships. System performance addresses whether the tool performs its stated function. Decision process outcomes address the patient’s understanding and participation. Relational outcomes address the quality of explanation, trust calibration, and the opportunity to disagree. Equity outcomes address subgroup performance and access to participation. Governance outcomes address traceability, response to unsafe output, and completion of follow-up. Increased trust should not be treated as an unqualified benefit because persuasive design or automation bias may increase trust without improving understanding. Table 4 summarizes the candidate measures and interpretive questions for evaluating AI-assisted shared decision making.

## 4. Discussion

### 4.1. Principal Contribution

This study developed a five-domain relational *framework* for artificial intelligence-assisted shared decision making. Its central contribution is to define success at the level of the decision rather than infer it from model performance, usability, or efficiency. A technically strong output supports shared decision making only when the patient can identify its role, clinicians can verify and contest it, preferences are expressed rather than presumed, uncertainty leads to a revisable plan, and accountability extends across the organization. The *framework* also clarifies the meaning of a triadic relationship. Artificial intelligence does not become an equal moral partner. The triad describes a practical change in the flow and authority of information. The patient and clinician remain the participants in deliberation, while the system can shape the content, sequence, and apparent certainty of that deliberation. The organization determines whether this influence is visible and governable.

The framework’s proposed originality lies in its operational organization of established safeguards for AI-assisted shared decision making. WHO guidance provides ethical principles and governance recommendations, while the EU AI Act establishes legal requirements, including human oversight provisions for high-risk AI systems within its scope [12,24]. Our framework connects these foundations to a specific unit of assessment: the clinical decision-making encounter and its supporting care pathway. For example, role and influence disclosure asks whether the patient can understand how AI materially affected the recommendation. Independent verification and contestability ask how clinically relevant output was checked and how disagreement can be addressed. Direct preference elicitation asks whether the patient’s expressed priorities, rather than inferred preferences alone, informed the plan. Actionable uncertainty and revisability connect limitations to reassessment and follow-up, while distributed accountability and continuity identify responsibility for subsequent action. The added contribution is the explicit linkage of each requirement to a relational question, a clinical test, a documentation or governance response, and candidate evaluation measures. These links make the proposed requirements available for observation and prospective testing; they do not establish their validity or effectiveness. The framework is therefore intended as a complementary operational proposal, not as a replacement for WHO guidance, a legal compliance instrument, or evidence that existing standards are ineffective.

### 4.2. Relation to Existing Evidence

The framework explains why positive perceptions in reviews do not establish better decisions. Patients may find an artificial intelligence-enabled decision aid easy to use while clinicians identify missing risk factors or outdated information [3]. A generative explanation may be readable while its citations are fabricated. An ambient scribe may reduce documentation time while introducing a clinically significant inaccuracy [4]. Each benefit remains relevant, but it requires a second question about how the benefit affects understanding, choice, and safety. The direct preference domain responds to a rapidly developing area of research. Artificial intelligence can extract and predict preferences from large datasets, electronic records, surveys, and digital traces [8]. These methods may help generate hypotheses or support research, particularly when preferences are otherwise difficult to capture. Their clinical use requires a stronger boundary. A predicted preference should prompt a question rather than settle a decision when the patient can speak. When the patient cannot participate, applicable law, advance directives, shared care planning, surrogate decision standards, and contextual interpretation remain essential. The ethical concerns identified for end-of-life decisions show how easily a social portrait can be mistaken for a person’s considered will [20]. The actionability domain is supported by recent qualitative evidence. Stakeholders in the 2026 interview study wanted to know not only the predicted risk but what action followed, who would explain the result, and who would respond if the prediction caused harm or distress [7]. These concerns fit the broader shared decision-making literature, which treats a decision as a process of option awareness, deliberation, and agreement rather than the delivery of information alone [1,15].

### 4.3. Implications for Clinical Practice

When a patient brings artificial intelligence-generated material to a consultation, clinicians can treat it as part of the informational history. The first questions concern the prompt, input data, complete answer, source, and action already taken or contemplated. The next question is what the patient found convincing or worrying. This approach separates the factual quality of the output from the need that led the patient to seek information. The clinician can retain correct material, identify the specific defect, add missing context, and explain how the revised understanding changes the options. When the healthcare organization supplies the system, the clinician should explain its function and material influence in concise terms that can be substantiated. A useful explanation might state that a tool estimated a defined outcome, that the inputs and meaning of the result were checked, and that one feature of the patient was poorly represented in the validation data. The conversation then returns to the choice: how the estimate changes the alternatives and which outcomes matter to the patient. Teach-back can test the clarity of the explanation without examining the patient. Asking the patient to describe the reason for the plan in their own words can reveal confusion about probabilities, alternatives, or follow-up. When disagreement persists, the clinician should identify whether it concerns facts, values, data use, distrust of automation, or the availability of a non-AI route. These concerns require different responses and should not be collapsed into a generic refusal of technology.

### 4.4. Legal and Professional Alignment

The relational *framework* aligns with the Italian model of informed consent while remaining conceptually applicable across jurisdictions. Law 219 of 2017 places consent within a relationship of care and trust in which patient autonomy meets professional competence, autonomy, and responsibility; it also recognizes communication time as care [22]. Law 132 of 2025 adds a right to information about the use of artificial intelligence in healthcare, prohibits discriminatory conditioning of access, and describes these systems as support while leaving the decision with health professionals [23]. These provisions do not reduce shared decision making to disclosure about technology. Information about artificial intelligence matters because it affects the patient’s understanding of the treatment, alternatives, risks, and professional reasoning. Article 7 of Law 132 [23] does not by itself establish a universal separate consent form for every use of artificial intelligence. The content and form of discussion depend on the system’s role, the materiality of its influence, clinical risk, data processing, and other applicable rules. Consent to care and the legal basis for processing health data also answer different questions. Italian data protection guidance notes that treatment strictly necessary for care under specified professional conditions does not depend on privacy consent, while other uses may require separate assessment [26]. The European Union AI Act contributes system-level duties where the high-risk regime applies, including risk management, data governance, technical documentation, logging, information for deployers, and human oversight [24]. Health software is not automatically high risk merely because it is used in healthcare; classification depends on intended purpose and the criteria in Article 6 and the relevant annexes. The relational *framework* does not replace these legal duties. It translates the information and organizational capacity they generate into questions that can be answered during care. International application would require adaptation to local legal requirements, healthcare organization, resources, and patient preferences regarding participation. The five domains can guide this assessment, while their implementation may differ: disclosure and documentation should reflect applicable consent and data protection rules; verification and escalation should follow local clinical pathways; and follow-up responsibilities should reflect how care is organized and funded. Direct preference elicitation should accommodate language, health literacy, and the patient’s desired involvement of family members or other trusted persons. In settings with limited digital access or technical capacity, assisted or non-digital alternatives and feasible verification arrangements require particular attention. The Italian and European instruments discussed here illustrate specific applications rather than universal legal requirements. Local stakeholder assessment and pilot implementation are needed to establish relevance and feasibility; cross-system applicability has not been demonstrated by this study.

### 4.5. Organizational Implications

Organizations should avoid assigning accountability to a clinician without providing the means to exercise it. Before deployment, they should define the intended use, target population, prohibited uses, local validation needs, data dependencies, expected user response, and escalation route. Training should cover both technical interpretation and relational communication. The simulation program reported by Kim and colleagues provides a useful educational model because participants practiced interpretation, explanation, option comparison, patient questions, feedback, and debriefing in the same exercise [16]. Monitoring should include more than technical drift. Organizations need to know whether outputs reach the right professional, whether patients see results before explanation, how often clinicians override the system, whether certain groups receive less usable information, and whether alerts lead to completed follow-up. Incident review should reconstruct the pathway rather than ask only whether a model was right or wrong. This approach is consistent with enterprise risk management, which connects prospective vulnerability assessment, incident analysis, continuous monitoring, and multidisciplinary oversight [17]. A practical starting point would be to pilot the five domains within one defined AI-assisted care pathway. Healthcare organizations could translate them into a brief encounter checklist and corresponding documentation fields, assign verification and follow-up responsibilities, and establish escalation routes for disputed or unsafe output. Clinicians could use the questions to explain AI’s material influence, review clinically relevant output, elicit patient priorities, and agree on reassessment arrangements. Policymakers could support implementation through procurement criteria, professional training, and evaluation requirements that include patient participation, accessibility, and continuity alongside technical performance. Initial implementation should assess feasibility, patient understanding, and documentation burden, with local adaptation before wider adoption. These are proposed implementation strategies, not interventions evaluated in this study.

### 4.6. Research Agenda

Prospective evaluation should match the stage of implementation. Early studies can test whether patients and clinicians correctly identify the system’s function, limits, and intended use. Simulation can examine verification behavior, communication of discordant results, and response to unexpected questions. Pragmatic trials can compare an artificial intelligence-assisted pathway with both usual care and a well-designed non-AI decision aid so that the incremental contribution of artificial intelligence is not confused with the effect of structured information. Study designs should follow the patient journey. Before the encounter, investigators can assess whether tools identify open decisions and prepare questions without creating false confidence. During the encounter, observation and validated measures can examine balanced option presentation, participation, direct preference elicitation, and the effect of output on the recommendation. After the encounter, studies can assess recall, adherence to the agreed plan, access to human follow-up, and understanding of reassessment triggers. Qualitative work remains necessary because inhibition caused by recording, perceived pressure from a score, or confusion created by conflicting explanations may not appear in technical metrics. A minimum reporting set for studies of artificial intelligence-assisted shared decision making should include the system function and version; intended user and clinical decision; target population and setting; alternatives presented; comparator; timing of patient access to output; professional review process; patient preference elicitation method; technical, decision, relational, equity, and safety outcomes; override and error handling; and follow-up responsibility. Subgroup analyses should be planned around clinically and socially relevant differences rather than added only after an overall effect is observed. The framework itself requires validation. Content validity can be tested with patients, clinicians, nurses, developers, ethicists, legal experts, and governance leaders. Feasibility studies can examine whether the five questions fit routine workflow. Construct validity would require evidence that higher adherence is associated with better comprehension, participation, value concordance, and continuity without unacceptable burden. Prospective studies should also test whether the standard identifies harms that existing usability and performance metrics miss. The five domains could be evaluated through indicators of patient understanding of AI’s influence, verification and handling of disagreement, elicitation of patient priorities, understanding of reassessment arrangements, and completion of assigned follow-up. These indicators could be assessed through consultation observation, patient interviews, and record review, building on Table 4. Definitions and assessment time points should be specified prospectively, and documentation alone should not be considered evidence of meaningful participation. A staged validation pathway could begin with a Delphi process involving patients, clinicians, nurses, AI developers, and experts in ethics and clinical governance to assess domain relevance, completeness, and clarity. A subsequent pilot implementation could examine feasibility, acceptability, documentation burden, and whether the proposed clinical tests can be applied consistently. Construct validity testing would then require predefined measures and hypotheses, such as whether greater adherence to the framework is associated with better patient understanding, participation, and concordance between expressed preferences and the agreed plan. These are proposed future studies; none were undertaken in the present work.

### 4.7. Strengths and Limitations

The study’s main strength is the integration of evidence, clinical practice, ethics, law, and governance into observable requirements. Structured eligibility criteria, source-type appraisal, a source matrix, discordant-case analysis, and explicit domain-retention rules make the analytical path inspectable. The framework also connects communication during the encounter to organizational conditions and measurable outcomes. Several limitations require emphasis. Structured purposive sampling is appropriate for conceptual framework development but is not an exhaustive systematic database search and may omit relevant studies, jurisdictions, technologies, or professional perspectives. No protocol was registered. The heterogeneous corpus could not support meta-analysis or a common risk-of-bias score, and evidence strength remains dependent on the design and limitations of the underlying sources. Framework development and coding were performed by a single author, so independent coding and inter-rater assessment were not available. Purposive selection may have favored sources consistent with the authors’ relational perspective, while the English- and Italian-language eligibility criteria may have limited the range of cultural and healthcare perspectives represented. Interpretation across empirical studies, conceptual analyses, and normative instruments also required judgments about how different forms of evidence should inform the framework. The resulting domains therefore reflect an interpretive organization of the selected material rather than categories uniquely determined by the sources. Alternative analysts or source selections might support different boundaries, additional domains, or a different treatment of cross-cutting themes. The domain-retention criteria make this reasoning explicit but do not eliminate interpretive subjectivity or establish the completeness of the five-domain structure. The legal discussion is illustrative and centered on Italian and European sources; it is not a substitute for case-specific legal analysis. Most empirical evidence concerns perceptions, feasibility, education, or selected implementations rather than patient outcomes. The proposed *framework* has not yet undergone stakeholder consensus methods or prospective clinical validation.

## 5. Conclusions

Artificial intelligence can strengthen shared decision making when it makes relevant information easier to verify, compare, and connect with a patient’s life. It can weaken the process when a precise score or fluent explanation closes discussion too early. The proposed relational *framework* keeps that distinction visible through five requirements: disclose the system’s function and material influence, verify clinically relevant output independently, elicit preferences directly, translate uncertainty into a revisable plan, and support distributed accountability with documentation and continuity. The proper measure of success is not whether a system appears intelligent or efficient. It is whether the patient can understand the proposal, influence it, and recognize how the decision can be revisited as circumstances or evidence change. Organizations must provide the information, training, authority, and monitoring that make this *framework* feasible. Prospective validation should now determine whether the framework improves decision quality, equity, safety, and follow-up across different clinical contexts.

## Figures and Tables

**Table 1:** Sources included in the qualitative framework synthesis.

Category	Source	Primary Analytical Contribution
Shared decision-making framework	Stiggelbout et al. 2015 [1]	Core stages and relational aims of shared decision making
Shared decision-making framework	Elwyn et al. 2017 [15]	Three-talk model linking team formation, option comparison, and decision deliberation
Evidence syntheses	Di Palma et al. 2025 [2] 39 publications	AI in collaborative care planning and shared decision making; heterogeneous designs and no pooled effect
Evidence syntheses	Hassan et al. 2024 [3] 26 articles	Patient and clinician perspectives on AI-enabled decision aids across screening, prevention, prognosis, and treatment
Evidence syntheses	Yang et al. 2025 [8] 67 studies	AI measurement and prediction of patient values and preferences; limited real-world validation and representativeness
Empirical studies	Lukac et al. 2025 [4] Pragmatic randomized trial	Documentation time, usability, and clinically significant inaccuracies of ambient AI scribes
Empirical studies	Benda et al. 2026 [7] 34 stakeholder interviews	Actionability, communication preferences, data quality, privacy, and shared governance
Empirical studies	Kim et al. 2026 [16] Pilot with 27 students	Simulation-based training in AI-assisted shared decision making; perceived competence and curricular needs
Conceptual and professional	Sauerbrei et al. 2023 [6]	Effects of AI on person-centred doctor-patient relationships
Conceptual and professional	Hack et al. 2026 [5]	AI-educated patient and hypothesized soft accountability
Conceptual and professional	Di Palma et al. 2025 [17]	Enterprise risk management for AI-related risks in healthcare
Conceptual and professional	Sokol 2026 [18]	Practical duties concerning system choice, confidentiality, verification, disclosure, documentation, and incidents
Conceptual and professional	Cheetham et al. 2025 [19]	Digital support across the patient journey and human factors in shared decision making
Conceptual and professional	Furlan et al. 2026 [20]	Ethical limits of AI in end-of-life decisions and risks of inferred wishes
Conceptual and professional	Penner et al. 2025 [21]	Informed consent, power, and nursing mediation in AI-supported maternity care
Legal and governance	Italian Law 219 of 2017 [22]	Consent within a relationship of care and trust; understandable information; communication time as care
Legal and governance	Italian Law 132 of 2025 [23]	Right to information about healthcare AI; professional decision authority; reliability and non-discrimination
Legal and governance	European Union AI Act [24]	Risk classification, documentation, information, logging, risk management, and human oversight where applicable
Legal and governance	WHO guidance on large multimodal models [12]	Autonomy, safety, transparency, accountability, inclusiveness, and equity
Legal and governance	FNOMCeO Code of Medical Ethics [25]	Professional judgment, communication, consent, safety, records, and information technology
Legal and governance	Italian Data Protection Authority 2019 [26]	Legal bases and conditions for processing health data in care

**Table 2:** Operational relational *framework* for artificial intelligence-assisted decisions.

Domain	Relational Question	Clinical Test	Record or Governance Response
Role and influence	Can the patient identify the system’s role and whether it changed the proposal?	Explain the function, contribution, influence, and case-relevant limitation	Record material AI influence when it affected the choice
Verification and contestability	Has clinically relevant output been checked independently and can it be challenged?	Check inputs, intended use, evidence, applicability, omissions, and discordant findings	Document why the output was accepted, modified, rejected, or escalated
Preferences	Did the patient’s expressed priorities alter the discussion?	Ask which outcomes matter, which burdens are least acceptable, and which constraints shape the choice	Record the preferences that materially influenced the plan
Uncertainty and revisability	Was uncertainty translated into a practical next step?	Define the outcome and time horizon; explain limits; agree on follow-up and triggers for reassessment	Specify unresolved information, timing, responsibility, and early review criteria
Accountability and continuity	Can the decision and the system’s contribution be reconstructed across the care pathway?	Use authorized systems, preserve confidentiality, follow escalation procedures, and report unsafe behavior	Maintain proportionate traceability, version information when required, and incident governance

**Table 3:** Relational risks and safeguards across the patient journey.

Stage	Typical AI Contribution	Relational Risk	Required Safeguard
Before the encounter	Patient-facing chatbot, portal explanation, automated triage, pre-visit decision aid	False confidence, fabricated sources, anxiety, digital exclusion, premature closure of options	Identify the decision as open; show source and limits; allow non-digital access; invite questions; specify urgent escalation routes
During the encounter	Prediction, classification, option comparison, image interpretation, ambient documentation	Automation bias, omitted alternatives, persuasive framing, misunderstood probabilities, inhibited candour	Disclose function and influence; verify inputs and output; compare options symmetrically; elicit preferences directly; confirm understanding
After the encounter	Generated summary, portal message, monitoring, follow-up recommendation, automated documentation	Mismatch with the agreed plan, privacy loss, unnoticed errors, unclear responsibility, lack of reassessment	Review generated content; document material reasoning; assign follow-up responsibility; monitor errors; keep a route to human review

**Table 4:** Candidate outcomes for evaluating artificial intelligence-assisted shared decision making.

Outcome Level	Candidate Measures	Interpretive Question
System performance	Accuracy, discrimination, calibration, missing data sensitivity, drift, clinically significant error rate	Does the system perform its intended technical function in this population and setting?
Decision process	Knowledge of options, risk comprehension, balanced option presentation, participation, decisional conflict, value concordance	Did the patient understand the choice and did expressed preferences influence the plan?
Relational quality	Ability to identify the AI role, opportunity to question output, clinician explanation, calibrated trust, perceived pressure	Did the technology support rather than close deliberation?
Equity	Proportion of eligible patients unable to access or use the AI-assisted pathway because of limited digital access, digital literacy, language, or accessibility barriers; uptake and completion rates stratified by these characteristics; availability and uptake of assisted or non-digital alternatives.	Who can use the pathway and whose information or preferences are underrepresented?
Safety and continuity	Overrides, near misses, adverse events, follow-up completion, time to reassessment, unresolved alert ownership	Can the plan be monitored and revisited when the situation or evidence changes?
Governance	Authorized use, version traceability, training completion, incident reporting, response time, corrective action	Can the organization reconstruct and improve the pathway?

## Data Availability

No new participant-level data were created or analyzed. The analytical corpus is identified in Table 1, and all sources are cited in the References Section.
